# Salutatory

**Published:** 1867-05

**Authors:** 


### EDITORIAL DEPARTMENT.

*Salutatory.*—In again making our bow to our friends of the Dental profession, after so long a silence, it is natural to cast an eye backward over the past.

When this Journal first started into existence, Dentistry presented a vastly different aspect from what it does now. Then it was just struggling into existence as a liberal profession. Men of ability and position were found in its ranks, but they had not leavened the entire mass. It is not to be disputed that the majority of practitioners of the art of Dentistry at that time had no claim to be considered members of a liberal profession. The men who under took the care of their neighbour's teeth were often very ill prepared for any study. The blacksmith of the village added dentistry to his other avocations, and even in cities, ingenious mechanics out of employment sought the practice of this art, as a convenient refuge from starvation.

The opportunities for these aspirants to gain any general knowledge of dental theory or art was exceedingly small. The student must enter the office of some dentist already engaged in business, and not better informed than his junior who was seeking for information. There he saw practice and hardened into all his master's errors. The art consisted of a collection of secrets which were jealously guarded in proportion to their inefficiency. Indeed there was an isolation among dentists more complete than any that existed among the different handicrafts. Under such a combination of unfortunate circumstances, it was simply impossible that a profession could exist.

Now, however, what do we see? The Dentists, as a profession, are recognized as Medical Specialists, just as much so as oculists and aurists. Socially they are gentlemen; professionally they command respect. They have a copious and creditable literature. They have pressed into their service the other sciences. Chemistry, Physiology, Pathology, Microscopy, Anatomy all contribute to this progress, and aid in the establishment of Dentistry on a sure foundation.

If we ask ourselves how this very desirable end has been brought about, we shall find that it has been due to a combination of causes. In the first place, the Colleges have contributed very largely to this result. They have, following the lead of the Baltimore College of Dental Surgery, taken instruction out of the hands of the office-practitioners. They have banished nostrums and secret practices, and have thrown open all the acquired knowledge in the possession of practitioners. They have trained a succession of students, year after year, in those studies which make up a liberal professional education. They have secured the services of men eminent in Medicine to teach their specialties, and have carried the study of these as far as is requisite for the most thorough training of the dentist. They have cultivated an *esprit de corps* and instilled into the minds of the pupils an abhorrence of the low arts and vulgar secrecy of the charlatan.

Nor can we who control the oldest periodical in the world devoted to the propagation of the principles upon which Dental Surgery is founded, forget the services rendered by the Journals. They have furnished continually, a medium by which members of the profession could communicate their ideas to their fellows. In this manner, information has been diffused and the habit of imparting it has been cultivated. Doubtless in these, as in all other Journals, literary, political or scientific, many worthless speculations have been published, but they have not been by any means destitute of valuable contributions to science both general and special. Thus Dentists have become known to each other all over the world; a current literature has been established, and the professional feeling generated by the Colleges has been fostered and strengthened. Need we say that, in the future, as in the past, we shall carefully cherish this *esprit de corps.*

There is still another agency which is too often overlooked, but which has been by no means inefficient in developing the resources of the profession. We allude to the Dental Depots. It is true they are commercial establishments, and their main object, as of all other trading concerns, is to make money for their owners. But what legitimate and honorable business ever failed to promote the general good? Every mercantile house created in a city is a new element of prosperity to the whole community. It brings business which employs many of the inhabitants. It introduces capital and increases the general wealth.

In the Dental Depot, we recognize more than this. It is a special and active agent in the improvement of the profession. The stimulus of competition induces each Depot to vie with its fellows in bringing out and laying before the profession, all new instruments and appliances of the art. The operator may there see at a glance the most recent improvements and suggestions. By this means a speedy trial is secured for any new proposition and it soon finds its proper level, by general adoption, if useful, by early rejection if valueless.

We have said nothing of Societies, because their action greatly resembles that of the Journals. They furnish a medium of communication, and accomplish generally the same ends as the periodicals, through which they make known their transactions.

